# Suspected Ovotesticular Disorders of Sexual Differentiation in a Phenotypic Male With Ambiguous Genitalia, Light Menstrual Flow, and Synchronous Bilateral Dysgerminoma: A Case Report From Ethiopia

**DOI:** 10.1155/crpe/7659991

**Published:** 2025-06-12

**Authors:** Melkamu Siferih, Tesfaye Negasa, Muluken Yifru, Adane Sisay, Genetu Tadele, Tajudin Adem, Mikias Gebrie, Worku Taye

**Affiliations:** ^1^Obstetrics and Gynecology, Debre Markos University, Debre Markos, Ethiopia; ^2^General Surgery, Medawolabu General Hospital, Dodola, Ethiopia; ^3^Radiology, St. Paul's Hospital Millennium Medical College, Addis Ababa, Ethiopia; ^4^Obstetrics and Gynecology, Arsi University, Asella, Ethiopia; ^5^Obstetrics and Gynecology, Jimma University, Jimma, Ethiopia; ^6^Pediatrics and Child Health, Addis Ababa University, Addis Ababa, Ethiopia; ^7^Pediatrics and Child Health, Debre Markos University, Debre Markos, Ethiopia; ^8^Clinical Midwifery, Debre Markos Comprehensive Specialized Hospital, Debre Markos, Ethiopia

**Keywords:** ambiguous genitalia, bilateral dysgerminoma, case report, disorders of sex development, Ethiopia, menstruation, ovotesticular DSD, phenotypic male

## Abstract

**Background:** Ovotesticular disorder of sexual differentiation (DSD) is one of the rarest congenital conditions affecting gonadal and sexual development, characterized by the coexistence of ovarian and testicular tissue within an individual. This condition often presents with ambiguous genitalia, atypical pubertal development, or unexpected menstrual activity. This case report details a 14-year-old phenotypic male with ambiguous genitalia, cyclic perineal bleeding, and synchronous bilateral dysgerminoma, underscoring the diagnostic complexities and management challenges encountered in resource-constrained settings.

**Case Presentation:** A 14-year-old individual assigned male at birth and raised as a boy presented with progressive abdominal distension, cyclic perineal bleeding, and absent male secondary sexual characteristics. Physical examination revealed ambiguous genitalia, a small phallic structure, a perineal opening with menstrual blood, and no palpable gonads. Hormonal analysis revealed elevated lactate dehydrogenase and gonadotropins, low testosterone levels, and increased estradiol. Imaging revealed an abdominopelvic mass highly suggestive of ovarian malignancy, and vaginal exploration confirmed Müllerian structures. Laparotomy revealed a 16-cm × 18-cm right adnexal mass, and histopathology confirmed dysgerminoma. The patient was lost to follow-up but returned 6 months later with a contralateral (left) adnexal mass, prompting oncologic referral.

**Conclusion:** Ovotesticular DSD with bilateral dysgerminoma is exceedingly rare and poses significant diagnostic and therapeutic challenges. Early diagnosis, multidisciplinary management, and timely oncologic intervention are crucial for optimizing patient outcomes, especially in resource-limited settings. This case underscores the critical need for heightened awareness, improved access to karyotyping, genetic and hormonal assessments, and long-term follow-up for individuals presenting with ambiguous genitalia and atypical pubertal development.

## 1. Introduction

Disorders of sex development (DSDs) represent a spectrum of congenital conditions characterized by atypical chromosomal, gonadal, and anatomical differentiation [1, 2]. Among these, ovotesticular DSD (OT-DSD) is an extremely rare genetic disorder, occurring in approximately one in 100,000 live births and comprising less than 10% of all DSD cases [3–6]. It is defined by the presence of both testicular and ovarian tissue within the same individual, often leading to ambiguous genitalia, atypical pubertal development, or unexpected menstrual function [7–10]. While 46, XX is the most common karyotype observed in OT-DSD, rare cases involving 46, XY or mosaic karyotypes have been reported [3, 6]. However, in many resource-limited settings, karyotyping and advanced genetic analyses are often unavailable, leading to clinical diagnosis based on phenotype, histopathology, and endocrine findings [4, 5, 11, 12].

Clinically, OT-DSD manifests with a broad spectrum of presentations, varying from ambiguous genitalia at birth to delayed or atypical progression of secondary sexual characteristics [4, 11, 13, 14]. Menstruation in a phenotypic male with ambiguous genitalia is an exceptionally rare phenomenon, suggesting the presence of functional ovarian tissue [10, 15, 16]. A major concern in DSD patients is the significantly increased risk of gonadal malignancy, particularly in individuals with Y chromosome material. Dysgerminoma, a germ cell tumor commonly associated with gonadal digenesis, is among the most frequently reported malignancies in DSD cases [17–19]. However, the occurrence of synchronous bilateral dysgerminoma in a suspected case of OT-DSD remains extraordinarily rare, with only a few cases reported globally [20–22].

Here, we present a unique case from Ethiopia: A phenotypic male with ambiguous genitalia who exhibited spontaneous menstruation and was found to have bilateral dysgerminoma, raising strong clinical suspicion for OT-DSD despite the unavailability of karyotyping. This case underscores the diagnostic and management challenges of DSD in resource-limited settings, highlights the oncologic risks associated with gonadal dysgenesis, and reinforces the need for multidisciplinary care in individuals with atypical sexual differentiation.

## 2. Case Presentation

A 14-year-old individual, assigned male at birth and reared as a boy, presented in February 2024 with worsening lower abdominal swelling and pain for one week, following a month-long history of progressive abdominal distension. A Grade 6 student from a remote area in Bale, Eastern Arsi Zone, Oromia region, sought care at Medawolabu General Hospital for persistent symptoms. Initially, the abdominal swelling was mild but significantly worsened over the past week, particularly along the midline below the umbilicus. The pain was dull, intermittent, and cramping, without associated fever, vomiting, or bowel habit changes.

Further history revealed that despite being raised as male, the patient had been menstruating for 2 years, though with reduced flow. Lifelong genital ambiguity was noted, characterized by a small phallic structure, an incompletely fused scrotum, and a perineal opening from which menstrual blood was observed. At puberty, male secondary sexual characteristics, such as voice deepening, facial hair growth, and increased musculature, failed to develop. Instead, the patient experienced progressive breast enlargement, absence of spontaneous erections or nocturnal emissions, and cyclical vaginal bleeding from the perineal opening. Additional symptoms included urinary dribbling, persistent abdominal distension, early satiety, occasional nausea, and unintentional weight loss, though there was no excessive body hair growth or signs of virilization.

Due to limited healthcare access and sociocultural barriers, the patient had never undergone medical evaluation for genital ambiguity. The patient had no prior history of hospitalization, surgical procedures, or chronic medical conditions and was delivered at full term at home without any noted perinatal complications or developmental delays. Family history was unremarkable for ambiguous genitalia, infertility, or intersex conditions, and no parental consanguinity was reported.

Raised in a Muslim Oromo household as a boy, the patient faced significant distress due to genital ambiguity and menstrual bleeding, leading to social isolation, stigma, and uncertainty about gender identity. The patient had not been in romantic or sexual relationships and was unaware of their reproductive potential.

**Figure 1:**
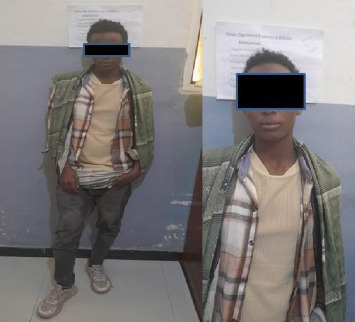
Photograph of a 14-year-old individual assigned male at birth.

**Figure 2:**
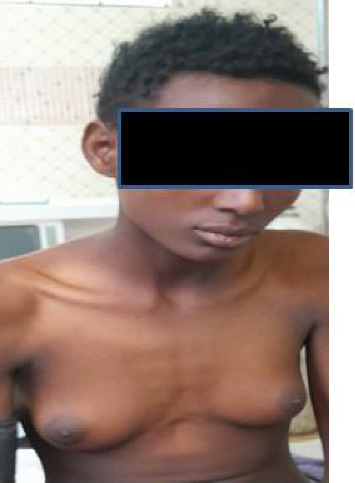
Photograph depicting breast development at Tanner Stage 4.

On physical examination, the patient appeared acutely ill but not in distress, with pronounced breast enlargement, rounded body contours, and a notable lack of male-pattern musculature. There were no signs of virilization, such as facial hair, excessive body hair, or voice deepening (Figures 1 and 2).

**Figure 3:**
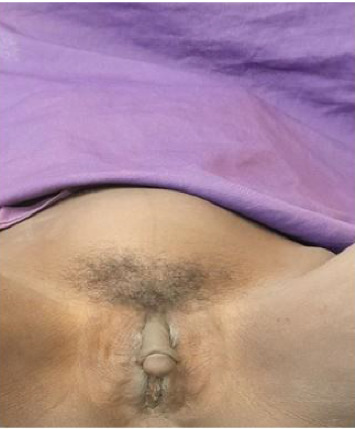
Photograph illustrating a 20-week abdominopelvic mass alongside ambiguous genitalia, characterized by clitoromegaly and partially fused labia majora.

Vital signs were stable, with normal heart rate, respiratory rate, blood pressure, and temperature. Abdominal examination revealed a distended abdomen with a firm, solid, irregularly contoured abdominopelvic mass, approximately the size of a 20-week gravid uterus. The mass was predominantly midline, extended into the lower quadrants, and was mildly tender but mobile in the transverse plane. It was nonadherent to the overlying skin, with no guarding, rebound tenderness, or palpable lymphadenopathy. The liver and spleen were not palpable, and there was no clinical evidence of ascites. Bowel sounds were present and normoactive. Genitourinary examination revealed ambiguous external genitalia, with a phallic structure resembling clitoromegaly rather than a fully developed male organ. The labioscrotal folds were incompletely fused, and a perineal opening was noted, with evidence of menstrual blood. No palpable gonads were detected in the labioscrotal folds, inguinal canal, or perineum. There were no urethral meatal abnormalities, overt urethral anomalies, or tenderness in the perineal or inguinal regions (Figure 3). Other systemic examinations, including cardiovascular, respiratory, musculoskeletal, and neurological assessments, were unremarkable.

Hematological and biochemical investigations, including complete blood count (CBC), renal and liver function tests, urinalysis, serum electrolytes (sodium and potassium), and random blood glucose (RBG), were all within normal reference ranges. Endocrine assessments, including thyroid-stimulating hormone (TSH), free T4, and cortisol, were also normal. Infectious disease screening for HIV provider-initiated testing and counseling (PITC), syphilis venereal disease research laboratory (VDRL), and hepatitis B surface antigen (HBsAg) yielded negative results. Tumor markers showed normal levels of β-hCG and alpha-fetoprotein (AFP), but lactate dehydrogenase (LDH) was elevated at 512 U/L (normal: 140–280 U/L). Hormonal analysis revealed low testosterone (35 ng/dL; normal: male 75–400 ng/dL, female < 10–50 ng/dL), elevated estradiol (122 pg/mL; normal: male < 10–40 pg/mL, female 15–200 pg/mL), and increased levels of follicle-stimulating hormone (FSH, 18 mIU/mL; normal: male 1–8 mIU/mL, female 1.5–10 mIU/mL), and luteinizing hormone (LH, 15 mIU/mL; normal: male 1–10 mIU/mL, female 0.5–15 mIU/mL). Anti-Müllerian hormone (AMH) was low at 10 ng/mL (normal: male 25–75 ng/mL, female < 5 ng/mL). A chest X-ray revealed no abnormalities. Karyotyping was not conducted due to financial limitations and the unavailability of necessary laboratory facilities.

**Figure 4:**
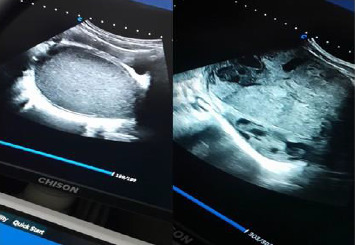
Abdominopelvic ultrasound revealing a large, heterogeneously hypoechoic mass displacing the right ovary.

Due to the inconclusive ultrasound findings (Figure 4) in distinguishing the mass from hematometra—especially considering the patient had experienced menstrual flow, albeit in a reduced amount, over the past two years—additional diagnostic evaluation was necessary. After obtaining explicit written consent and assent, the initial surgical plan involved vaginal exploration through a small perineal opening located just below the urethral meatus. Upon vaginal entry, speculum examination revealed a visible cervix. To assess patency, the cervix was probed toward the uterine cavity, confirming that the abdominal mass was unrelated to the endometrial cavity. Consequently, an ovarian origin of the mass was established.

**Figure 5:**
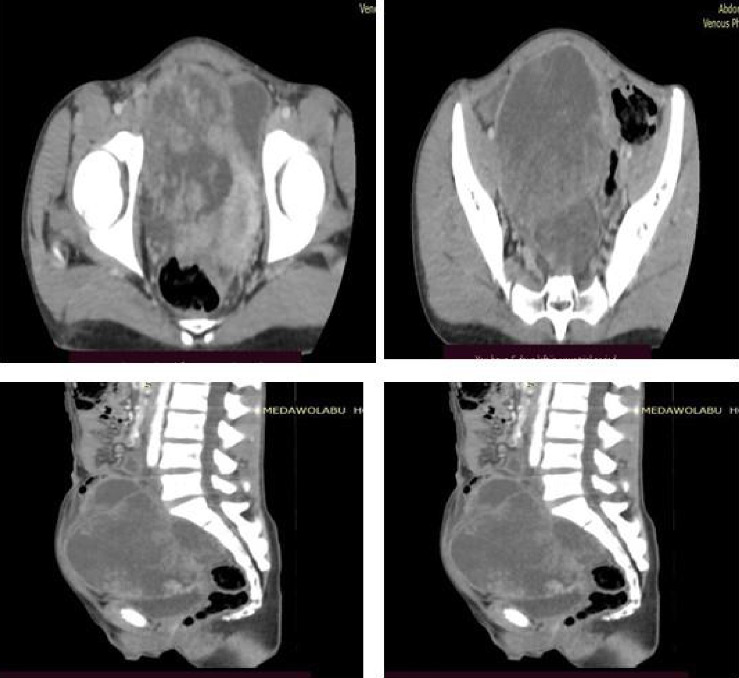
Contrast-enhanced computed tomography (CECT) scan demonstrating a giant, heterogeneously enhancing right adnexal mass with a strong suspicion of malignancy.

A contrast-enhanced computed tomography (CECT) scan of the abdomen and pelvis, performed with multiplanar reconstruction (MPR) at a 3-mm slice thickness, revealed a large, lobulated, predominantly solid mass in the right adnexal region with heterogeneous postcontrast enhancement. Nonenhancing hypodense areas within the lesion suggest necrosis, and the mass exerts a mass effect, displacing the uterus toward the left. These findings indicate a giant, heterogeneously enhancing right adnexal mass with a high suspicion of malignancy. Differential diagnoses include dysgerminoma and mixed germ cell tumors (Figure 5).

**Figure 6:**
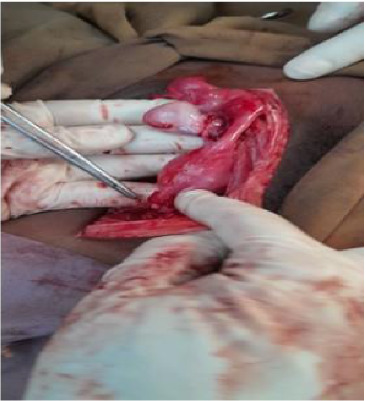
Intraoperative photograph displaying internal female reproductive structures.

As the patient's abdominal pain worsened, an exploratory laparotomy was performed through a midline vertical incision, revealing a large mass adherent to the bowel but without ascites. Intraoperatively, a general surgeon was consulted. The uterus was normal in appearance and of pediatric size, while the left adnexa, including the ovary and fallopian tube, showed no abnormalities. On the right side, a fragile 16-cm × 18-cm adnexal mass was identified, necessitating a unilateral salpingo-oophorectomy along with peritoneal and omental biopsies and peritoneal washing (Figure 6). Hemostasis was secured, and the abdomen was closed in layers. The patient remained stable throughout the 10-day hospitalization. Postoperatively, the patient was counseled on intermittent vaginal dilator use and subsequently began experiencing regular monthly menses.

Histopathological analysis confirmed dysgerminoma, revealing a solid, lobulated mass with a gray-white to tan cut surface, focal areas of necrosis and hemorrhage, and large polygonal tumor cells with clear cytoplasm, prominent nucleoli, fibrovascular septa with dense lymphocytic infiltration, and scattered mitotic activity. No ovarian or testicular structures were identified.

Despite counseling on the need for further oncologic evaluation, the patient was lost to follow-up for 6 months. The patient later returned with a 2-week history of abdominal swelling and pain. A follow-up ultrasound revealed a 5-cm × 6-cm solid mass in the left adnexa, highly suggestive of synchronous dysgerminoma. The patient was promptly referred to an oncology center for comprehensive staging and chemotherapy, but the subsequent management and treatment outcomes remain unknown.

## 3. Discussion

DSDs encompass a spectrum of congenital conditions in which chromosomal, gonadal, or anatomical sex development is atypical. OT-DSD, formerly termed true hermaphroditism, is among the rarest forms, characterized by the presence of both ovarian and testicular tissue in the same individual. This case presents a unique and diagnostically challenging scenario of a 14-year-old individual assigned male at birth who exhibited ambiguous genitalia, menstrual-like bleeding, and synchronous bilateral dysgerminoma, a rare malignant germ cell tumor typically arising in patients with gonadal dysgenesis.

The patient's lifelong ambiguous genitalia, absence of virilization at puberty, and cyclical perineal bleeding strongly indicated an underlying DSD [23, 24]. The presence of a visible cervix during vaginal exploration, combined with cyclic perineal bleeding, suggests functional Müllerian structures. This finding is consistent with OT-DSD or other forms of gonadal dysgenesis [25, 26]. Increased gonadotropin levels (FSH and LH), coupled with low testosterone and elevated estradiol, provide further evidence of primary gonadal failure or dysgenesis, a characteristic finding in these cases. Consistent with prior findings [27–30] on DSD, this hormonal profile indicates a disrupted gonadal environment, resulting in impaired steroidogenesis. However, unlike studies [30–32] documenting partial testicular function in some OT-DSD cases, this patient exhibited complete testicular failure at the time of evaluation, suggesting a more severe degree of gonadal dysgenesis and insufficient androgen production. Despite this, the presence of ambiguous genitalia implies that there were some degrees of androgen exposure during early fetal development, likely due to transient testicular function before eventual gonadal failure [24, 33, 34]. Alternatively, mosaicism or mixed gonadal tissue may have contributed to the observed phenotype, where regions of functional testicular tissue coexisted with dysgenetic gonads, producing variable androgen levels [35, 36]. Additionally, adrenal-derived androgens could have played a role, though insufficient for full virilization [37]. This pattern is in agreement with earlier reports [24, 38, 39] in which individuals with severe gonadal dysgenesis exhibit incomplete masculinization due to early but insufficient androgen action.

Differential diagnoses in adolescents with DSD presenting with abdominal masses include hematometra, gonadoblastoma, dysgerminoma, Sertoli–Leydig cell tumors, and mixed germ cell tumors [40]. Hematometra was initially considered plausible due to the history of light cyclical bleeding. However, intraoperative findings ruled out this diagnosis due to the absence of endometrial distension. Unlike cases where hematometra occurs in the presence of fully developed Müllerian structures [41], this patient lacked sufficient Müllerian development to support such a diagnosis. Elevated LDH in this patient played a crucial role in diagnosing ovarian dysgerminoma and narrowing differential diagnoses. As a key tumor marker, LDH is frequently elevated in dysgerminoma due to its high metabolic activity and rapid proliferation [42, 43]. This finding in our patient reinforced LDH's role as a sensitive biomarker for disease detection and progression.

Dysgerminomas are the most common malignant germ cell tumors associated with gonadal dysgenesis, occurring in approximately 5% of individuals with DSD [18, 20, 22]. Recent studies [44–46] report up to 15% of dysgerminomas present with bilateral involvement, highlighting the risk of residual gonadal tissue undergoing malignant transformation. In contrast to prior cases and related studies [47–51] where dysgerminomas are detected early, this patient presented with advanced disease and a rapid recurrence, suggesting an aggressive tumor behavior. The pathophysiology of dysgerminoma in the context of OT-DSD likely involves an abnormal gonadal microenvironment that predisposes to incomplete differentiation and tumorigenesis [20, 52]. Unlike patients with classic gonadal dysgenesis, where gonadoblastoma is a well-established precursor to dysgerminoma in many DSD cases [53, 54], this tumor manifested as pure dysgerminoma without gonadoblastoma components, suggesting an alternative malignant pathway and highlighting the variability in dysgerminoma pathogenesis among DSD patients.

One of the perplexing aspects of this case was the inability to detect residual gonadal tissue despite the presence of Müllerian structures and hormonal evidence suggestive of dysgenetic gonads [32, 55]. Possible explanations include gonadal atrophy or fibrosis resulting from prolonged gonadal dysgenesis, rendering the tissue undetectable during surgical exploration. Consistent with previous reports [27, 56] describing gonadal regression due to insufficient hormonal support, this patient may have experienced spontaneous resorption of gonadal structures. Another plausible explanation is that dysgerminoma development led to complete replacement of gonadal tissue. Additionally, anatomical displacement or ectopic localization of residual gonadal structures could have affected their detectability. The absence of ovarian or testicular tissue may also reflect sampling bias—if only tumor-dense regions were analyzed, residual gonadal tissue might have been present but excluded from examined sections. More extensive tissue sampling or immunohistochemical studies may be required to detect remnants of ovarian or testicular differentiation. Moreover, extensive tumor necrosis or regression could obscure the original gonadal architecture, further complicating histopathological identification.

Optimal management of dysgerminoma involves surgical resection, with chemotherapy indicated for advanced or recurrent disease [57, 58]. In our case, surgery aimed to confirm the tumor's origin while preserving reproductive structures. However, the synchronous nature and rapid recurrence necessitated early oncologic intervention. The patient's loss to follow-up underscores a common challenge in resource-limited settings, where specialized oncology care is often inaccessible. Unlike early-stage cases managed with surgery alone [59], adjuvant chemotherapy was warranted due to the recurrence [60]. While genetic and karyotypic evaluation is crucial for DSD classification [3, 6], its absence in this case limits definitive categorization. In contrast to high-resource settings, where molecular and cytogenetic analysis informs management [61], diagnosis in low-resource regions relies on clinical and biochemical assessments [62]. Moreover, many African countries, including Ethiopia, lack structured national protocols for the long-term follow-up of DSD patients, posing significant challenges to patient retention and ongoing clinical surveillance [63].

Psychosocial considerations play a crucial role in DSD management, particularly in gender assignment decisions and long-term psychological well-being [64]. Consistent with studies [65, 66] highlighting the influence of societal and cultural norms on gender identity, this patient's upbringing as a male despite ambiguous genitalia underscores the significant role of societal expectations. While high-income settings offer greater access to open discussion and specialized care for gender dysphoria, resource-limited regions often face barriers such as stigma and inadequate psychosocial support, which can hinder gender identity development and well-being [67, 68]. Ethical challenges related to gender identity disclosure and decision-making in adolescents add complexity to management, underscoring the importance of multidisciplinary input from endocrinologists, geneticists, and mental health professionals [64, 69].

This study faces a significant limitation because karyotyping services are not accessible in the country even though chromosomal analysis could elaborate on dysgerminoma pathogenesis. Attempts to acquire both original and digital histological slides failed due to logistical and administrative challenges so an independent pathological examination became impossible. The lack of karyotyping services combined with the difficulties acquiring original histological slides restricted thorough diagnostic assessment so it could impact the results' reproducibility.

## 4. Conclusion

Managing OT-DSD with synchronous bilateral dysgerminoma presents significant diagnostic and therapeutic challenges, particularly in settings with limited resources. Unlike the typical clinical course of dysgerminomas, the rapid recurrence in this case underscores the crucial need for early detection, continuous oncologic monitoring, and timely medical intervention. These findings further highlight the importance of early genetic and oncologic assessment, a structured multidisciplinary approach, and standardized long-term follow-up to improve patient outcomes. Future efforts should focus on expanding access to genetic testing, developing national guidelines for DSD management, and integrating psychosocial support services to enhance the quality of life for affected individuals.

## Data Availability

All data supporting the findings of this case report are fully presented within the article. Further details may be provided by the corresponding author upon reasonable request, subject to ethical and confidentiality considerations.

## Nomenclature

AFP: Alpha-fetoprotein
AMH: Anti-Müllerian hormone
CBC: Complete blood count
DSD: Disorder of sex development
FSH: Follicle-stimulating hormone
HBsAg: Hepatitis B surface antigen
hCG: Human chorionic gonadotropin
LDH: Lactate dehydrogenase
LH: Luteinizing hormone
OT-DSD: Ovotesticular disorder of sex development
PITC: Provider-initiated testing and counseling
RBG: Random blood glucose
TSH: Thyroid-stimulating hormone
VDRL: Venereal disease research laboratory
